# Health care in rural areas: proposal of a new telemedicine program assisted from the reference health centers, for a sustainable digitization and its contribution to the carbon footprint reduction

**DOI:** 10.1016/j.heliyon.2022.e09812

**Published:** 2022-06-28

**Authors:** Moncho-Santonja Maria, Aparisi-Navarro Silvia, Defez Garcia Beatriz, Davol Andrew, Peris-Fajarnés Guillermo

**Affiliations:** a Centro de Investigación en Tecnologias Gráficas. Universitat Politècnica de Valencia, Valencia, Spain; b California Polytechnic State University San Luis Obispo, CA, USA

**Keywords:** Rural telemedicine, Pollutants, Sustainable digitization, Carbon footprint, Atmospheric emissions

## Abstract

**Introduction:**

This paper studies and quantifies the environmental benefits of implementing a new telemedicine service for users of the public health system in a rural area of Alicante (Spain). The proposed telemedicine service is based on carrying out 20% of the follow-on consultations with a specialist virtually from the Reference Health Centres with the support of qualified staff. This way of providing medical care remotely will be a good transition to fully online medical services, especially for the elderly. The proposed model avoids the displacement of users to the Alcoy Hospital, reducing the distances to be travelled, which will be directly reflected in a reduction of the emission of pollutants (carbon footprint) generated by patients' vehicles.

**Methods:**

Data from the Alcoy Health Department were used for 2019, the last year of normal activity of the health centres before Covid-19. Using data from the Department's health management report and the emission factors of the vehicles, we calculated the distances, hours, litres of fuel saved, as well as the tonnes of CO_2_ equivalent, CO_2_, CH_4_ and N_2_O.

**Results:**

With the implementation of this type of telemedicine, journeys would be avoided, saving 447,279 km, 7,580 h and 38,019 L of fuel. The emission into the atmosphere of 79.26 metric tons of CO_2_, 74.5 kg of CH_4_ and 487.28 kg of N_2_O per year would be avoided.

**Conclusions:**

The implementation of this telemedicine service contributes to a high degree to: (a) increasing the environmental sustainability of the rural health sector thanks to the reduction of traffic emissions (saving 9% of pollutants compared to the current system), (b) decongesting the health system by reducing face-to-face visits to specialists, (c) increasing the quality of life of patients by avoiding road travel (d) promoting the digitalisation of the rural population.

## 1 Introduction

User access to the health care system depends to a large extent on the distance and time patients spend traveling to visit a doctor. Methods and technologies are now available to minimise geographical barriers and increase the accessibility of health services. Telemedicine is one of the most effective ways of increasing the accessibility of services and its implementation is increasingly necessary in rural areas, where access to specialists can be difficult (Lipszyc et al., 2020; Maciel et al., 2020).

The benefit of the implementation and use of telemedicine is not only for the users of health services (both patients and health workers) but also for the environment (Cardier et al., 2016; Connor et al., 2019; Miah et al., 2019; Wootton et al., 2010).

Despite being aware of the benefits and advantages of the use of Information Communication Technologies (ICTs) in the health sector, there are still areas where telemedicine is not yet established, either due to technical, economic, organisational or end-user acceptance barriers (Harst et al., 2020).

The use of videoconferencing and telephonic modalities to provide healthcare has increased exponentially since the pandemic caused by Covid-19 (de Guzman et al., 2021), with videoconferencing taking on particular relevance (Cain et al., 2017). The increase has been mainly in populations living in cities and whose access to ICT use is fully established.

In rural and ageing populations, there is a need to promote the use of these new modalities. Given the notable and growing ageing of the population in rural areas (Elizalde-San Miguel and Díaz-Gandasegui, 2016) and the scarce use of ICTs by a large part of the inhabitants of small municipalities (“INE. Instituto Nacional de Estadística,” n. d.), telemedicine, understood as attendance at medical consultations via videoconferencing from home, may not be the best way of obtaining healthcare in rural areas, especially for the elderly. Therefore, the implementation of telemedicine in an assisted manner is being considered. In other words, telemedicine from a Reference Health Centre (RHC), where the patient receives technical assistance and is guided during the telematic consultation. It is crucial to support patients (especially older patients) in the use of new technologies to ensure their successful implementation and that they do not fall into disuse.

This case study is of a rural area in the interior of the province of Alicante (Spain). We find there the Health Department of Alcoy. This department covers a total of 32 municipalities with wide demographic dispersion, with more than half of the population (60.7%) concentrated in two municipalities (Alcoy and Ibi) and the rest distributed in municipalities of less than 1,000 inhabitants for the most part.

**Figure 1:**
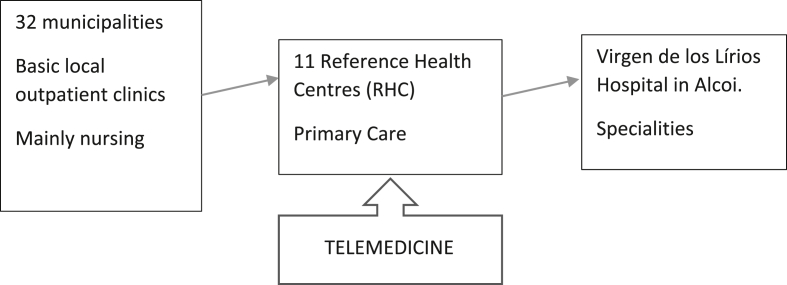
Structure and functioning of the alcoy health department.

The structure and functioning of the Health Department is shown in Figure 1. In most of the municipalities, there is an outpatient clinic where mainly nursing work is carried out. For primary care, the patient must go to a Reference Health Centre (RHC), each RHC serves several municipalities. And finally, for specialist consultation and diagnostic tests, users must go to the Virgen de Los Lirios Hospital (VLH) located in Alcoy.

The area covered by the Alcoy Health Department has a rugged terrain, with many mountain roads, which makes it difficult to travel. In addition, the territory has a higher population ageing rate (132.95%) (de Salut D’alcoi, 2018)than the national average (120.5%) (de Salut D’alcoi, 2018)Population ageing rate is defined as the percentage of the population over 64 years of age out of the population under 16 years of age on 1 January of a given year.

The implementation of a telemedicine service in each of the RHC (not in municipal outpatient clinics) would be a feasible solution for rural areas with a dispersed and ageing population. Patients could consult with a specialist in the RHC, much closer to their municipality, accompanied and guided by outpatient health staff.

**Figure 2:**
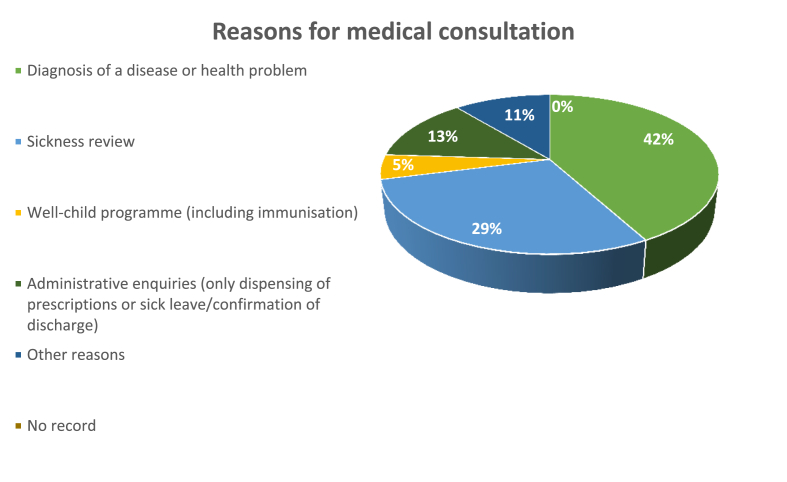
Reasons for medical consultation. Source: Data from the national institute of statistics (INE).

In Spain 42% (13% + 29%) of visits to the doctor's surgery are for administrative consultations, monitoring of illnesses, collection of results, prescriptions, etc (“INE. Instituto Nacional de Estadística,” n. d.) (Figure 2). In many of these cases, the consultation could also be carried out telematically, avoiding unnecessary trips to the hospital.

In this study, we are going to evaluate the implementation of a telemedicine service for carrying out speciality medical consultations from reference health centres. It has been considered that 100% of the first visits to speciality consultations at the Virgen de los Lirios Hospital (VLH) should be carried out in person, so that the specialist can carry out a direct examination of the patient. However, it has been considered that (at least) 20% of the follow-on consultations with the specialist are for the collection of results and periodic reviews of chronic diseases, which can be carried out telematically from the RHC (Figure 2).

The assumption that part of the follow-on visits to specialists will be made from the RHCs is motivated by several factors. Firstly, a large portion of the population is elderly and does not make use of ICTs, therefore, visits from patients' homes are ruled out. Secondly, local outpatient clinics are not open every day, which makes it difficult to fit in saturated speciality schedules. Thirdly, there is not always a doctor to be found in local clinics. In Spain, only doctors are able to renew prescriptions, report results, etc. This is in addition to the need for regular monitoring of the ageing population by a primary care doctor. Therefore, it is proposed not to implement telecare in local outpatient clinics, but in the reference health centres where there are always primary care doctors.

Given this scenario, the study quantifies the atmospheric pollutants associated with unnecessary journeys to the hospital that occur in real and usual clinical practice in the area. This will show the effect that the implementation of a telemedicine programme such as the one proposed will have in terms of reducing the carbon footprint in rural areas.

## 2 Materials and methods

The study used data from the Public Administration and estimated the benefit of implementing the telemedicine service from the point of view of reducing the number of trips to the hospital. The savings associated with this reduction in terms of economic benefit, time and emissions have been quantified.

The data on the number of visits made per inhabitant and municipality to the specialist was obtained from the Management Report of the Regional Ministry of Universal Health and Public Health for 2019 (“Memoria, 2019 - Conselleria de Sanitat Universal i Salut Pública,” n. d.)

**Figure 3:**
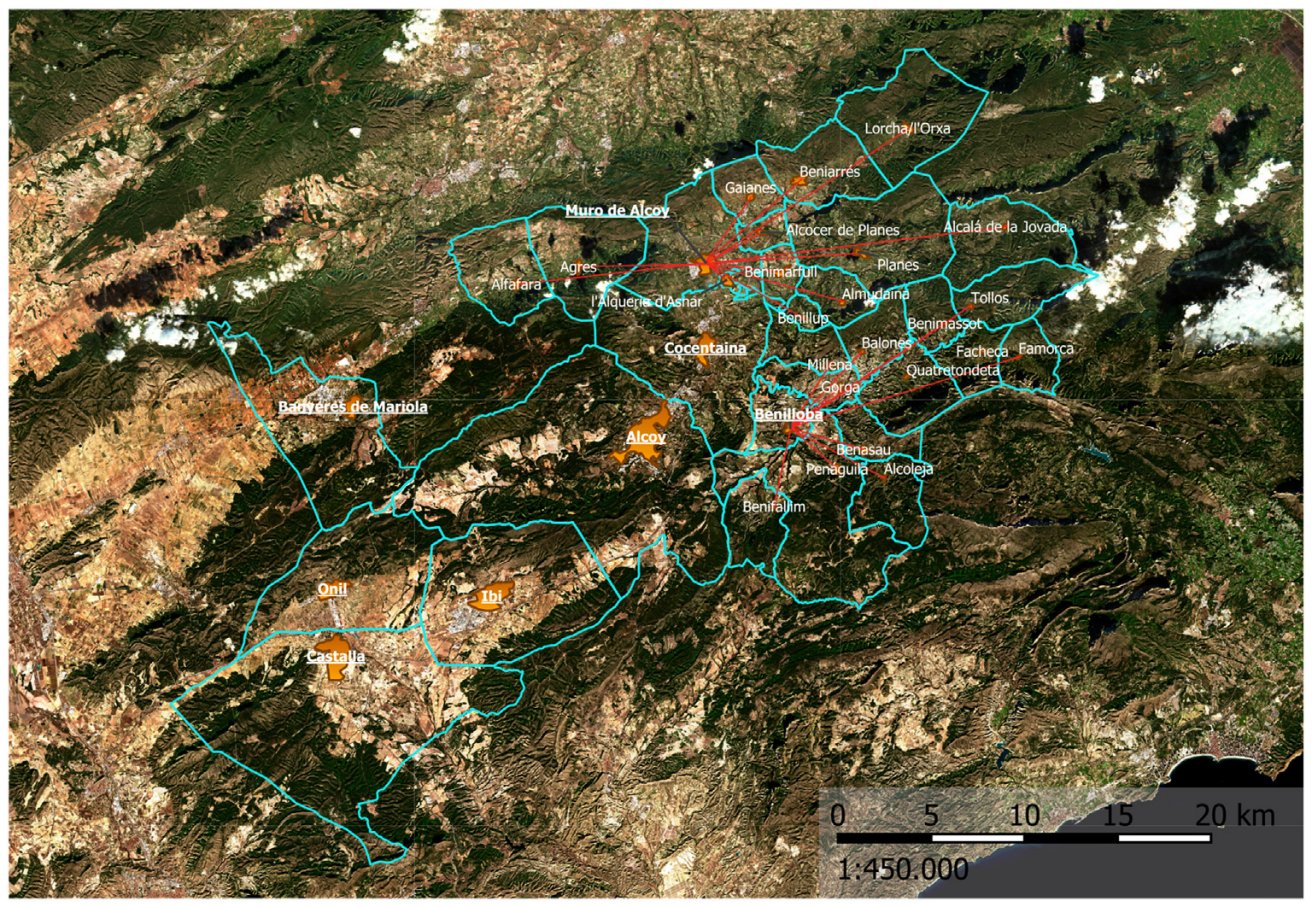
Municipalities and their Reference Health Centres. The lines link the municipalities with their RHC. If there is no line, it means that the RHC is in the same municipality.

In the Alcoy Health Department, all speciality consultations are carried out at the Virgen de los Lirios Hospital in Alcoy. The Department works as follows: a patient goes to his or her family doctor in the RHC (Figure 3) and is referred to the specialist. This first appointment with the specialist takes place at the VLH, which is located at an average distance of 20.33 km from each municipality. For follow on visits to the specialist, patients must return to the same hospital.

In the scenario we propose, it has been considered that 20% of these follow-on visits can be carried out telematically from a RHC, which are located at an average distance of 7.6 km from each municipality. As can be seen, the distance to be covered in these follow-on visits is reduced by almost a third. This reduction in the distance travelled is associated with a reduction in emissions of atmospheric pollutants by transport vehicles.

Of the total number of consultations made by specialists, it has been considered that their population distribution corresponds to the distribution and percentage of demographic occupation of the municipalities in the region, obtained from the National Institute of Statistics (INE) (“INE. Instituto Nacional de Estadística,” n. d.)

The ratio of follow-on visits to specialists (0.9613%) was obtained from the Management Report of the Regional Ministry of Universal Health and Public Health for 2019 (“Memoria, 2019 - Conselleria de Sanitat Universal i Salut Pública,” n. d.) (Table 1) and the number of first and follow-on visits to specialists per municipality was calculated using data from the INE.

**Table 1:** Outpatients consultations Department of Health of Alcoy, year 2019.

Outpatient consultations in 2019				
Health department	Consultations			Relation
	First	Follow-on	Total	Follow-on/First
Alcoy	112075	107742	219817	0,9613

The number of follow-on visits was evaluated assuming a telemedicine service was implemented and the number of visits to the hospital reduced by at least 20%.

The Google Maps tool was used to calculate the distance and travel time with the option "fastest route with regular traffic" both for the calculation of the distance travelled from each municipality to the VLH and for the calculation of the distance travelled from each municipality to its RHC.

For the calculation of the fuel consumption associated with each journey, an average vehicle consumption of 8.5 L/100 km was considered for both diesel and petrol vehicles. For the calculation of the price of the journey (1), the average prices for the year 2019 consulted in the Electronic Headquarters of the Spanish Ministry for Ecological Transition and Demographic Challenge were taken into account. The distribution of the type of vehicle found in the province of Alicante in terms of fuel type is 51.41% petrol vehicles and 48.59% diesel vehicles.

(1) Averagefuelprice=(petrolprice∗%ofpetrolvehicles)+(dieselprice∗%dieselvehicles)

In one case, distance, times, litres of fuel, euros of fuel, carbon dioxide, methane and nitrous oxide emissions have been calculated without the implementation of the telemedicine system. In the other case, the same variables were calculated if 20% of follow-on visits to specialists were made from the RHC. In both scenarios (with and without telemedicine implementation), the calculation of air pollutant emissions was obtained by multiplying the kilometres travelled (round trip) by the emissions per km. The emissions per km and pollutant are shown in Table 2.

**Table 2:** UK government GHG conversion factors for company reporting (“Greenhouse gas reporting: conversion factors 2019 - GOV.UK,” n.d.).

POLLUTANTS	Kg CO_2_ e	Kg CO_2_	Kg CH_4_	Kg N_2_O
Diesel Car (Kg/km)	0,17336	0,17152	0,00000432	0,00184
Petrol Car (Kg/km)	0,18084	0,18014	0,00032	0,00038
Average per km (Kg/km)	0,17720542	0,17595149	0,000166609	0,001089423

The savings in pollutants, time, distance, litres of fuel and euros by implementing the telemedicine system in the RHCs were obtained by subtracting each of the variables calculated for each of the scenarios.

## 3 Results

As shown in Table 3, if 20% of the follow-on visits to the specialist had been made from the Reference Health Centres during 2019, a total of 21,548 visits to the Hospital would have been avoided.

**Table 3:** Number of consultation to the specialist.

No. of visits per year	219817
No. of first visits per year	112075
No. of follow-on visits per year	107742
No. possible annual Tele assistance	21548
No. of follow-on face-to-face visits per year	86194

In total, the completion of these 21,548 visits from the RHCs represents a mileage saving of 447,279km. This translates into an average of 20.7 km per visit or a saving of 21 min of travel time per visit. Or, in other words, a total of 7580 travel hours per year. In total, a fuel reduction of 38,010 L is obtained, which represents an economic saving of more than 47,800 euros (see Table 4).

**Table 4:** Savings in travel distance, fuel time and costs.

SAVINGS	round trip	
Distance	447279	km
time	7580	h
fuel	38019	liters
euros fuel	47838	€

The emissions of atmospheric pollutants that would be avoided can be seen in Table 5. In total, 79 metric tons of carbon dioxide, 74.52 kg of methane and 487.27 kg of nitrous oxide could be avoided per year. For each specialist consultation carried out by the RHCs, emissions of 3.678 kg of CO_2_ equivalent (3.652 kg CO_2_, 3.45 g of methane and 22.6 g of nitrous oxide) would be avoided.

**Table 5:** Reduction of pollutant gases.

	Total Kg	Average Journey (Kg)
Carbon dioxide equivalent CO_2_ e	79260,27	3,678
Carbon dioxide CO_2_	78699,41	3,652
Methane CH_4_	74,52	0,0034
Nitrous oxide N_2_O	487,27	0,0226

By performing 20% of the follow-on visits from the RHC, 9% of the air pollutants emitted during patient travel would be saved compared to the current system of face-to-face consultation.

## 4 Discussion

This study has analysed the environmental benefits and savings that could be obtained by carrying out 20% of the follow-on visits to the specialist telematically from the reference health centre, in terms of time, distance, fuel use and cost, as well as the reduction of greenhouse gas emissions. Given the characteristics of the study area (rural, with long distances to travel) we see that telemedicine can be a good solution to improve accessibility to specialist doctors. In addition, taking into account the ageing rate of the Health Department, telemedicine from the reference health centre can facilitate the reception of telecare services in this Department.

This type of telematic assistance with support in the health centres could perfectly well be replicated in rural areas with similar characteristics to the health department studied, for example in the health departments of Requena, Xátiva, Elda or Orihuela. All of these departments provide care to a similar volume of patients to the case studied, and with exactly the same functioning as described in this study. The suitability of the use of telemedicine in these departments could be explained, as in the case study, by the mountainous orography of the area as well as by the population distribution or the number of health areas and care centres as can be seen in the table below.

**Table:** 

Health Department	Outpatient consultations 2019	Health areas	Reference Health Centers	Basic Clinics
Alcoi (case study)	219987	10	11	30
Requena	98690	5	5	41
Xativa Ontinyent	307792	17	19	52
Elda	318480	9	12	10
Orihuela	282816	7	7	29
Elx	258828	6	6	7

In general it can be stated that the proposed telecare is suitable for rural areas with an ageing population and rugged terrain in Spain and in other countries. However, a case-by-case study would be preferable to determine the suitability of this type of telecare in each area.

In this study, the health department has a total population of 135842 inhabitants. Patients have to travel to the hospital in Alcoy if they need to be seen by a specialist. In 20% of the cases, the follow-on visits are for reasons of revision of chronic conditions, revision of tests, collection of prescriptions, etc. If, on these occasions, the patient went to the RHC, the patient could obtain the same service, but reducing the journey and the emission of atmospheric pollutants.

However, there are other benefits of using telemedicine. It facilitates the attendance of patients' relatives (Østervang et al., 2019), among others.

The results of this work are consistent with those found in the 2019 study (Vidal-Alaball et al., 2019) which studied greenhouse gas reductions for teledermatology consultations in a rural region of Spain. This study calculates a reduction for an average journey of 21.3 km of 3248g of CO_2_.

A recent study (Blenkinsop et al., 2021) calculates the distance saved by telemedicine services in the epilepsy speciality and determines that for a distance of 224000 km, between 35000-40000 kg of carbon dioxide equivalent are saved, very much in line with the data obtained in this study.

In another study (Paquette and Lin, 2019), for an average one-way distance of 15 km and an average travel time of 19.5 min, vascular surgery patients managed to save 3.78 euros per visit by carrying out the consultation telematically, as well as reducing emissions of 1,632 kg of carbon dioxide for a total of 146 medical visits.

We can see from other studies that telematic consultation eliminates or reduces patient travel, which saves both travel time and associated costs (Abbott et al., 2018; Beck et al., 2017; Humer and Campling, 2017).

A more detailed study by speciality will help to determine which pathologies and which specialities are the most suitable for telematic consultations. On this occasion, variables related to patient travel to the hospital have been studied, although there are factors such as lost working hours, waiting times or parking costs that would justify the implementation of the system even more. A broader study will provide a tool to help in the generalisation of telemedicine as an efficient way of medical consultation.

In the present study, the private car was assumed to be the vehicle of transport, without taking into account public transport, since the supply in this area is very limited (Vidal-Alaball et al., 2019). The percentage of avoidable follow-on visits has been assumed based on data on consultations from the national statistics institute (INE), however, a study with semi-structured interviews with specialist doctors has helped to give the final focus to the study.

## 5 Conclusions

This study confirms that the implementation of a telemedicine service in rural areas for medical specialties would have an environmental benefit in terms of reducing the emission of air pollutants. It would also save time and money for patients, as well as improve their quality of life.

Telemedicine programmes should be considered as an option in the global strategy to reduce greenhouse gas emissions in the fight against climate change: the implementation of telemedicine is seen as a modality that significantly reduces the pollution caused by traffic associated with medical consultations.

The implementation of a telemedicine service:

- improves the quality of life of patients and accompanying persons: It reduces the time patients have to spend on the road either driving or being accompanied to the doctor's surgery. In turn, virtual health consultations can facilitate assistance to patients' relatives.
- is a start in the digitalisation of the elderly population in rural environments.
- decongests the health system by reducing the number of face-to-face visits to the specialist's surgery.
- If a joint municipal transport system is implemented in each Reference Health Centre, individual patient journeys are reduced and it avoids either elderly people driving on the road or their companions having to leave their jobs during working hours to accompany them.
- reduces the number of patients in the Hospital, avoiding the saturation of the facilities, a crucial factor especially in times such as the current pandemic.
- increases the environmental sustainability of the health sector thanks to the reduction of traffic emissions (saving of 9% of pollutants compared to the current system).

## Declarations

### Author contribution statement

M Moncho-Santoja & S Aparisi-Navarro: Performed the experiments; Analyzed and interpreted the data; Contributed reagents, materials, analysis tools or data; Wrote the paper.

B Defez García: Conceived and designed the experiments; Analyzed and interpreted the data.

A Davol: Analyzed and interpreted the data; Wrote the paper.

G Peris-Fajarnés: Conceived and designed the experiments.

### Funding statement

Silvia Aparisi-Navarro was supported by Universitat Politècnica de València [PAID-01-2020].

Maria Moncho-Santonja was supported by Conselleria d'Educació, Investigació, Cultura i Esport [ACIF-20].

### Data availability statement

Data will be made available on request.

### Declaration of interests statement

The authors declare no conflict of interest.

### Additional information

No additional information is available for this paper.
